# Longitudinal Associations Between Sexual Regulatory Focus and Sexual Health and Well-Being

**DOI:** 10.1007/s10508-026-03427-z

**Published:** 2026-04-18

**Authors:** David L. Rodrigues, Richard O. de Visser

**Affiliations:** 1 Iscte-Instituto Universitário de Lisboa, CIS-Iscte, Av. das Forças Armadas, Bldg. 4, Room A211, 1649-026 Lisbon, Portugal; 2 Department of Primary Care & Public Health, Brighton and Sussex Medical School, Falmer, UK

**Keywords:** Sexual regulatory focus, Sexual health, Sexual well-being, Condom use, Sexual satisfaction

## Abstract

Sexual health and well-being (SHWB) encompasses physical and emotional aspects beyond disease absence. Research has shown that sexual decisions and behaviors are informed by predominant motives for pleasure or safety, with some individuals prioritizing health protection and risk avoidance (i.e., predominant focus on prevention) and others sexual pleasure and rewards (i.e., predominant focus on promotion) with casual partners. This longitudinal study with individuals in Spain and Portugal explored how sexual regulatory focus was related to sexual responses, behaviors, and experiences with casual partners at baseline (T1, *N* = 811) and three months later (T2, *N* = 527). Results of a latent profile analysis revealed three distinct profiles. Participants predominantly focused on prevention reported higher sexual health outcomes at baseline (e.g., more sexual inhibition due to risk awareness; more condom use in different sexual activities) and three months later (e.g., enacted safer sexual activities) to the detriment of their sexual well-being (e.g., less sexual satisfaction). In contrast, participants predominantly focused on promotion reported higher sexual well-being outcomes at baseline (e.g., more sexual excitation; more sexual communal strength) and three months later (e.g., more sexual satisfaction; more autonomous reasons for having sex) potentially to the detriment of their sexual health (e.g., enacted riskier sexual activities; but were also more likely to have been tested for sexually transmitted infections). A third group of participants with a dual focus managed to protect their sexual health (e.g., enacted safer sexual activities later on) without compromising their sexual well-being (e.g., more sexual excitation; more sexual satisfaction later on). These findings show that sexual regulatory focus is a crucial aspect to consider in efforts aimed at fostering SHWB.

**Supplementary Information:**

The online version contains supplementary material available at 10.1007/s10508-026-03427-z.

## Introduction

Sexual health and well-being (SHWB) includes physical and emotional aspects of well-being that go beyond the absence of diseases (Mitchell et al., 2021). This comprehensive view highlights the importance of having positive interactions in sex (e.g., sexual pleasure) while being respectful of others (e.g., consent to sexual activity) and aware of potential health risks (e.g., sexually transmitted infections [STIs]). However, individuals often value certain facets of SHWB to the detriment of others when making decisions and behaving in casual sex encounters. This is likely determined by the ability to self-regulate feelings and actions according to predominant needs and goals in a given context (Baumeister & Vohs, 2007). The regulatory focus theory (Higgins, 2015) is a particularly relevant framework that can help understand why some people decide to engage in certain behaviors despite potential health risks. Across multiple domains, individuals with a predominant focus on promotion are motivated by opportunities and excitement, and are more likely to take risks in pursuit of gains, whereas individuals with a predominant focus on prevention are motivated by safety and stability, and are more likely to avoid potentially adverse outcomes in riskier situations (Zou & Scholer, 2016).

In the sexuality domain, research has shown that individuals have different priorities as a result of their predominant sexual regulatory focus. For example, individuals predominantly focused on promotion (vs. prevention) report being more sexually satisfied with casual partners (Evans-Paulson et al., 2022; Rodrigues et al., 2024a, 2024b, 2024c) and score higher on risk-taking traits, including greater comfort with casual sex (i.e., sociosexually unrestricted) and lower sexual restraint (Rodrigues et al., 2019). Not only are these individuals quicker to discard the need for condoms in hypothetical scenarios (Rodrigues, 2023) and perceive more barriers to condom use (e.g., condoms decrease intimacy in sex; condoms are uncomfortable to use; Rodrigues et al., 2024a, 2024b, 2024c), but they also engage in condomless sex more frequently (Rodrigues et al., 2020). Still, individuals predominantly focused on promotion (vs. prevention) are not oblivious to their risk-taking behaviors—they acknowledge a higher susceptibility to infections and get tested for STIs more frequently (Rodrigues et al., 2022, 2023). Taken together, these findings suggest that individuals predominantly focused on promotion tend to prioritize their sexual well-being (i.e., pursuing pleasure and rewards in casual sex encounters), even at the risk of immediate negative health outcomes. In contrast, individuals predominantly focused on prevention tend to prioritize their sexual health (i.e., avoiding potential infections from casual sex encounters), even at the cost of limiting their sexual pleasure with casual partners.

Extending past research, we argue that differences in sexual regulatory focus are also likely to shape individuals’ typical responses to sexual cues and interactions with casual partners, and their sexual health practices and experiences of sexual well-being over time. The dual control model (Janssen & Bancroft, 2023) proposes that sexual responses and arousal are dependent upon the activation and balance of two systems. The excitation system is related to sexual activity and pleasure—similar to being more focused on promotion—, whereas the inhibition system is related to performance or threat concerns—similar to being more focused on prevention. Particularly relevant to our study, individuals with a stronger predisposition for sexual excitation (and weaker predisposition for sexual inhibition) are more easily sexually aroused, experience more sexual desire and satisfaction, have more sexual partners, and take more risks in casual sex (Bancroft et al., 2009; Janssen & Bancroft, 2007; Velten, 2017). Hence, having a predominant focus on the promotion of pleasure or the prevention of threats may predispose individuals toward distinct patterns in the activation of each system in casual sex encounters.

In the context of romantic relationships, individuals experience greater sexual desire and satisfaction when they meet their partners’ sexual needs (i.e., sexual communal strength; Muise et al., 2013), and those who engage in sexual activity with their romantic partners for more autonomous reasons (e.g., to pursue sexual pleasure and intimacy) and less controlled reasons (e.g., out of pressure and obligations) experience greater sexual desire and satisfaction over time (Shoikhedbrod et al., 2023). Plus, being sexually satisfied with romantic partners also predicts positive affective reactions later on (Debrot et al., 2017). Exploring these dynamics in the context of casual sex encounters, individuals whose behavior is more centered around both personal and joint sexual satisfaction (i.e., predominantly focused on promotion; Evans-Paulson et al., 2022; Rodrigues et al., 2024a, 2024b, 2024c) may be more inclined to experience higher sexual communal strength with casual partners, endorse more autonomous (and less controlled) reasons to have casual sex, and experience more positive (and less negative) affect with their casual partners. In contrast, individuals whose functioning is more centered around threat awareness and control over condom use (i.e., predominantly focused on prevention; Rodrigues et al., 2019, 2022) may be more inclined to communicate about sexual health with their casual partners (Noar et al., 2006). These individuals may also perceive greater health benefits over time, given that enacting safer sexual behaviors is linked to better perceived physical health (Samuolis & McGeorge, 2020).

### Current Study and Hypotheses

We conducted a pre-registered longitudinal study (https://osf.io/nr3s5) with individuals living in Spain and Portugal to examine if sexual regulatory focus determined sexual responses, behaviors, and experiences with casual partners. Instead of using the variable-centered approach detailed in the pre-registration document, we adopted a more sophisticated person-centered approach in this study. Specifically, we conducted a Latent Profile Analysis (LPA) to establish profiles based on patterns of co-occurring promotion and prevention motives in sexuality, and assign participants to a given profile with a specific degree of probability. This allowed us to examine in greater detail the potential nuances that can arise from assessing the construct on two separate (but correlated) motives in sexuality, instead of relying on correlations or a priori categorizations (for a similar argument, see Rodrigues et al., 2024a, 2024b, 2024c; Rodrigues & Guedes, 2025). Importantly, this analytic approach did not change the hypotheses outlined in the pre-registration document. At baseline (T1), we expected individuals predominantly focused on prevention to report more sexual inhibition (H1a), whereas individuals predominantly focused on promotion were expected to report more sexual excitation (H1b) and sexual communal strength (H1c). To replicate past research, we conducted additional exploratory analyses at baseline to examine sexual regulatory focus differences in sexual health practices (i.e., condomless sex; ST testing; sexual health routine checkups).

Over time, we expected sexual regulatory focus scores at T1 to predict those same scores three months later (T2), supporting the predictive validity and temporal reliability of the measure (H2). We also expected individuals predominantly focused on prevention at T1 to have engaged in condomless sex less frequently with casual partners (H3), experience more negative (and less positive) affect when recalling condomless sex encounters (H4), endorse more sexual health communication with their casual partners (H5), and report better sexual health (H6) at T2. In contrast, individuals predominantly focused on promotion at T1 were expected to endorse more autonomous (e.g., excitement; sexual desire) and fewer controlled (e.g., pressure) reasons for having casual sex (H7), report more sexual satisfaction with casual partners (H8), and be more likely have been tested for STIs (H9) at T2. Whenever possible, we explored differences between oral and penetrative sex, as oral sex is perceived to carry fewer health risks than intercourse and is more frequently practiced without using condoms (Ballester-Arnal et al., 2022; Strome et al., 2022). Lastly, we explored potential differences between participants in Spain and Portugal.

## Method

### Participants and Procedure

Data were collected as part of the Prevent2Protect project (https://osf.io/rhg7f) approved by the Ethics Council at Iscte-Instituto Universitário de Lisboa (Reference: 70/2021). Using a conservative approach to power analysis, our pre-registered estimation for a structural equation model with a moderate effect size of *R*^2^ = .20 (Cohen, 1988) and 90% power recommended a minimum sample size of 678 participants. This estimation was increased by 20% to account for longitudinal attrition, resulting in a target sample of 813 participants.

Clickworker registrants from Spain and Portugal were invited to participate in a study on sexuality and sexual behavior. To be eligible, participants had to be at least 18 years old, reside in Spain or Portugal, have engaged in casual sex activity, and be single or in casual relationships. Participants who failed to meet these inclusion criteria were redirected to the end of the survey and thanked for their interest. Eligible participants who completed the survey at T1 received €3 in their account and three months later received an invitation to participate in the follow-up survey (T2). Invitations were sent through the platform and no identifying information was shared with the researchers. Participants who completed the survey at T2 received €5 in their account.

A total of 831 participants completed the survey at T1, and 560 participants completed the survey at T2. At each time point, we included two attention check items (e.g., “Please select the option “3”. This is not a trick question”), and asked participants to indicate how attentive they were while completing the survey (1 = *No attention*, 2 = *Very little attention*, 3 = *Moderate amount of attention*, 4 = *Very close attention*) and if they wanted to retain their responses for analyses (1 = *I want to maintain my responses* or 2 = *I want to remove my responses and prevent them from being analyzed*). Participants who failed at least one of the attention checks (*n*_T1_ = 11, *n*_T2_ = 23), who indicated they paid little attention to the questionnaire (responses < 2; *n*_T1_ = 9, *n*_T2_ = 6), and who wanted their responses withdrawn (*n*_T2_ = 4) were excluded.

Participants (*N*_T1_ = 811, *N*_T2_ = 527) were, on average, 33 years old, most identified as White, identified as heterosexual, were living in metropolitan areas, and were working. Around half identified as women, resided in Spain, had a university degree, and were managing on their current income. Table 1 shows detailed characteristics for each time point. Despite a high attrition rate between time points (35%), comparisons revealed no significant demographic differences between T1 and T2 samples, all *p* ≥ .240.

**Table 1:** Demographic characteristics

	T1 (*N* = 811)	T2 (*N* = 527)
	*M* (*SD*) or *n* (%)	*M* (*SD*) or *n* (%)
*Age* (min = 18, max = 75)	33.56 (10.01)	33.28 (9.58)
*Ethnic background*		
Arab	5 (0.6)	4 (0.8)
Asian	1 (0.1)	1 (0.2)
Black	46 (5.7)	24 (4.6)
Latinx	124 (15.3)	73 (13.9)
Mixed race	8 (1.0)	6 (1.1)
Romany	1 (0.1)	1 (0.2)
White	616 (76.0)	412 (78.2)
Prefer not to answer	10 (1.2)	6 (1.1)
*Gender*		
Man	391 (48.2)	241 (45.7)
Transgender or gender non-binary	14 (1.8)	10.0 (1.9)
Woman	403 (49.7)	274 (52.0)
Prefer not to answer	3 (0.4)	2 (0.4)
*Sexual orientation*		
Asexual	6 (0.7)	5 (0.9)
Bisexual	128 (15.8)	91 (17.3)
Heterosexual	634 (78.2)	404 (76.7)
Lesbian/Gay	29 (3.6)	19 (3.6)
Pansexual	11 (1.4)	7 (1.3)
Queer	3 (0.4)	1 (0.2)
*Country*		
Spain	402 (49.6)	279 (52.9)
Portugal	409 (50.4)	248 (47.1)
*Residence*		
Metropolitan area	509 (62.8)	333 (63.2)
Rural area	106 (13.1)	68 (12.9)
Suburban area	194 (23.9)	125 (23.7)
Prefer not to answer	2 (0.2)	1 (0.2)
*Completed education*		
Primary or secondary school	11 (1.4)	7 (1.3)
High school	259 (31.9)	162 (30.7)
University degree	356 (43.9)	222 (42.1)
Post-graduate (Master’s; PhD)	163 (20.1)	122 (23.1)
Professional course	21 (2.6)	13 (2.5)
Prefer not to answer	1 (0.1)	1 (0.2)
*Occupation*		
Retired	10 (1.2)	7 (1.3)
Stay-at-home parent	9 (1.1)	4 (0.8)
Student (part or full-time)	175 (21.6)	120 (22.8)
Unemployed	81 (10.0)	54 (10.2)
Working (part or full-time)	535 (66.0)	341 (64.7)
Prefer not to answer	1 (0.1)	1 (0.2)
*Socioeconomic status*		
Difficult to live on current income	253 (31.4)	150 (28.6)
Managing on current income	387 (48.0)	261 (49.8)
Living comfortably on current income	155 (19.2)	105 (19.9)
Prefer not to answer	11 (1.4)	8 (1.5)
*Relationship status*		
Casually dating one person	165 (20.3)	107 (20.3)
Casually dating multiple people	316 (39.0)	222 (42.1)
Single without a relationship	330 (40.7)	198 (37.6)

### Measures

Measures already validated in both countries—Regulatory Focus in Sexuality, Sexual Inhibition and Sexual Excitation Scales, Positive and Negative Affect Schedule, and New Sexual Satisfaction Scale—were subject to confirmatory factor analyses (CFA). Following recommendations (Byrne, 2012), we examined absolute (*χ*^2^; SRMR) and relative fit indices (TLI), non-centrality indices (CFI; RMSEA), and standardized regression paths (λ) between items and their factor. Measures that were not validated in at least one country—Sexual Communal Strength, Reasons to Have Sex, and Sexual Health Communication—were subject to Exploratory Factor Analyses (EFA) with principal axis factoring and promax rotation. Items with factor loadings > .40 in more than one factor and items with factor loadings < .40 in all factors were removed until a final solution was reached. Reliability was computed using McDonald’s omega (*ω*; Hayes & Coutts, 2020). Analyses were conducted with JASP (JASP Team, 2024) and results are summarized in the Supplementary Materials (see also Tables 2 and 5 for correlations between subscales).

**Table 2:** Descriptive statistics and correlations for measures at T1

	*M* (*SD*)	1	2	3	4	5	6	7	8	9	10
1. Prevention focus (1–7)	4.86 (1.51)	–									
2. Promotion focus (1–7)	5.02 (1.90)	− .17^***^	–								
3. Sexual excitation (1–5)	3.13 (0.78)	− .31^***^	.31^***^	–							
4. Sexual inhibition: Performance failure (1–5)	2.54 (0.83)	− .10^**^	− .00	.04	–						
5. Sexual inhibition: Sexual risk threats (1–5)	3.51 (0.93)	.13^***^	− .08^*^	− .12^***^	.34^***^	–					
6. Sexual communal strength (1–5)	3.65 (0.60)	− .03	.17^***^	.20^***^	− .11^**^	− .08^*^	–				
7. Condomless oral sex (1–7)	5.07 (2.41)	− .17^***^	.18^***^	− 13^***^	− .03	− .10^**^	.13^***^	–			
8. Condomless vaginal sex (1–7)	3.59 (2.22)	− .15^***^	.09^*^	.08^*^	.09^*^	− .01	.07	.33^***^	–		
9. Condomless anal sex (1–7)	2.79 (2.18)	− .29^***^	.14^***^	.13^***^	.08^*^	− .04	.05	.29^***^	.51^***^	–	
10. Frequency of STI testing (1–7)	2.60 (1.83)	− .23^***^	.11^***^	.07^*^	.05	− .02	− .10^**^	− .00	.02	.11^**^	–
11. Frequency of routine checkups (1–4)	2.29 (0.92)	− .08^*^	.11^**^	− .05	.14^***^	.08^*^	− .27^***^	− .03	− .08	.01	.40^***^

Note: Mean score range is displayed between brackets

***p ≤ .001, **p ≤ .010, *p ≤ .050

#### Sexual Regulatory Focus (T1 and T2)

We used the Regulatory Focus in Sexuality scale, previously validated in Spain (Rodrigues, 2022) and Portugal (Rodrigues et al., 2019), to assess prevention (three items; e.g., “Not being careful enough in my sex life has gotten me into trouble at times”) and promotion motives in sex (six items; e.g., “I am typically striving to fulfill my desires with my sex life”). Responses were given on 7-point rating scales (1 = *Not at all true of me* to 7 = *Very true of me*) and averaged for each subscale. Higher scores indicated a stronger focus on prevention (*ω*_T1_ = .67; *ω*_T2_ = .76) and promotion (*ω*_T1_ = .84; *ω*_T2_ = .85). Subscale scores were negatively correlated within time points, both *p* < .001.

#### Sexual Activity and Sexual Health Practices (T1)

As part of the inclusion criteria, participants indicated if they ever had oral sex (1 = *No* or 2 = *Yes*), vaginal sex (1 = *No* or 2 = *Yes*), or anal sex (1 = *No* or 2 = *Yes*) with casual partners. For each “*Yes*” response, participants also indicated how frequently they tend to have condomless sex with casual partners (each item: 1 = *Rarely* to 7 = *Every time I have sex*). Participants were also asked to indicate how frequently they get tested for STIs (1 = *I have never been tested* to 7 = *I get tested frequently*) and attend routine sexual health checkups (1 = *I have never been to one*, 2 = *Less than once a year*, 3 = *About once a year*, 4 = *More than once a year*).

#### Sexual Inhibition and Sexual Excitation (T1)

We used the Sexual Inhibition/Sexual Excitation Scales—Short Form (SIS/SES-SF; Carpenter et al., 2020), previously validated in Spain (Sierra et al., 2024) and Portugal (Quinta Gomes et al., 2018), to assess participants’ typical responses in sex. We assessed sexual excitation (SES; six items; e.g., “When I start fantasizing about sex, I quickly become sexually aroused”), sexual inhibition due to performance failure threats (SIS1; four items, e.g., “I cannot get aroused unless I focus exclusively on sexual stimulation”), and sexual inhibition due to the perception of sexual health threats (SIS2; four items, e.g., “If I realize there is a risk of catching a sexually transmitted infection, I am unlikely to stay sexually aroused”). Responses were given on 5-point rating scales (1 = *Strongly disagree* to 5 = *Strongly agree*) and averaged for each subscale. Higher scores indicated more sexual excitation (*ω* = .73) and sexual inhibition (*ω*_SIS1_ = .64, *ω*_SIS2_ = .69). SIS2 scores were positively correlated with SIS 1, *p* < .001, and negatively correlated with SES, *p* < .001.

#### Sexual Communal Strength (T1)

We used the Sexual Communal Strength Scale (Muise et al., 2013) to assess how attentive participants typically are to the sexual needs of casual partners (six items; e.g., “How far would you be willing to go to meet your casual partner’s sexual needs?”). Responses were given in 5-point rating scales (1 = *Not at all* to 5 = *Extremely*) and averaged. Higher scores indicated more sexual communal strength (*ω* = .67).

#### Sexual Activity and Affective Reactions to Condomless Sex (T2)

Using three separate items, participants indicated if they had oral, vaginal, or anal sex with casual partners since they last participated in the study (i.e., over the past three months). Responses to each item were given on a dichotomous scale (1 = *No* or 2 = *Yes*). Participants who had casual sex (i.e., “*Yes*” responses) also indicated how frequently they had condomless sex (e.g., “How often did you have oral sex without using condoms in the last three months?”; responses given on a 7-point rating scale from 1 = *Never* to 7 = *Every time I had sex*) and with how many partners (e.g., “With how many partners did you have oral sex without using condoms in the last three months? Please write the number”; open-ended responses). Responses for condomless oral sex (*M*_frequency_ = 5.86, *SD*_frequency_ = 1.83; *M*_partners_ = 1.84, *SD*_partners_ = 1.62), vaginal sex (*M*_frequency_ = 4.44, *SD*_frequency_ = 2.50; *M*_partners_ = 1.61, *SD*_partners_ = 1.62), and anal sex (*M*_frequency_ = 4.16, *SD*_frequency_ = 2.58; *M*_partners_ = 2.36, *SD*_partners_ = 5.60) were then standardized to ensure the same measurement scale and then averaged within each sexual activity. Higher scores indicated a higher likelihood of enacting riskier sexual activities over the past three months (i.e., more frequent condomless sex with a higher number of partners). Participants who had condomless sex activities (i.e., responses ≥ 2) were additionally asked to think about their most recent condomless sexual encounter and indicate how intensely (1 = *Not at all* to 5 = *Extremely*) they experienced positive affect (four items, e.g., “Excited”) and negative affect (five items, e.g., “Guilty”), using the items from the internationally reliable Positive and Negative Affect Schedule Short-Form (I-PANAS-SF; Thompson, 2007). The original PANAS had already been validated in Spain (Lopez-Gomez et al., 2015) and Portugal (Galinha & Pais-Ribeiro, 2005). Responses for each subscale were averaged, with higher scores indicating more positive (*ω*_Oral_ = .82; *ω*_Vaginal_ = .80; *ω*_Anal_ = .85) and negative affect (*ω*_Oral_ = .81; *ω*_Vaginal_ = .80; *ω*_Anal_ = .80). Subscale scores were negatively correlated for each sexual activity, all *p* < .001.

#### Reasons to Have Sex (T2)

We used Shoikhedbrod et al.’s (2023) version of the Sexual Motivation Scale (Gravel et al., 2016) to assess the extent to which participants had sex over the past three months for autonomous (four items, e.g., “I engaged in sex to pursue my own pleasure”) and controlled reasons (three items, e.g., “Because I didn’t want to be criticized by my partner”). Responses were given in 7-point rating scales (1 = *Not at all* to 7 = *Completely*) and averaged for each subscale. Higher scores indicated more autonomous (*ω* = .56) and controlled (*ω* = .69) reasons for having had casual sex. Unexpectedly, subscale scores were uncorrelated, *p* = .569.

#### Sexual Satisfaction (T2)

We used the New Sexual Satisfaction Scale—Short Form (Štulhofer et al., 2010), previously validated in Spain (Strizzi et al., 2016) and Portugal (Pechorro et al., 2016), to assess how sexually satisfied participants were with themselves (six items; e.g., “The quality of my orgasms”), and the joint sexual activities with casual partners (six items; e.g., “The variety of my sexual activities”) considering the sexual encounters they had over the past three months (1 = *Not at all satisfied* to 7 = *Extremely satisfied*). We averaged responses for each subscale, with higher scores indicating more sexual satisfaction with oneself (*ω* = .87) and sexual activities (*ω* = .85). Subscale scores were positively correlated, *p* < .001.

#### Sexual Health Communication (T2)

We used the Health Protective Sexual Communication Scale (Catania, 2020) to assess how frequently participants discussed health protection with casual partners over the past three months (1 = *Never* to 7 = *Every time I had sex*) using eight items (e.g., “How often did you ask your casual sex partners how they felt about using condoms before having sex”). Responses were averaged, with higher scores indicating more sexual health communication (*ω* = .85).

#### Sexual Health (T2)

We assessed sexual health practices using two separate items. We adapted the physical health item from the Short-Form Health Survey (Ware et al., 1996) and asked participants “Overall, how would you rate your sexual health?” (1 = *Poor* to 7 = *Excellent*). We also asked participants “Did you get tested for STIs in the last three months? (e.g., HIV, chlamydia, gonorrhea, syphilis)” (1 = *No* or 2 = *Yes*).

### Analytic Plan

As pre-registered, we computed descriptive statistics and overall correlations for each time point. However, instead of testing our hypotheses using correlations and structural equation modeling with separate sexual regulatory focus scores, we computed an LPA in Mplus (Muthén & Muthén, 2017) to establish sexual regulatory focus profiles. To determine the number of latent profiles, we compared model fit indicators by examining Akaike’s Information Criterion (AIC), Bayesian information criterion (BIC), sample size–adjusted BIC (SABIC), and the *p* values of both the Lo, Mendell, and Rubin test (LMR) and the bootstrapped likelihood ratio test (BLRT). Following standard recommendations (Spurk et al., 2020), we identified the model with the best fit based on lower AIC, BIC, and SABIC values, and significant LMR and BLRT results, entropy levels (values ≥ .80 indicate lower classification uncertainty), and profile distribution percentages. Because participants were classified into distinct sexual regulatory focus profiles, we revised the hypothesis testing strategy detailed in the pre-registration document to be consistent with profile-based comparisons. Specifically, we computed ANOVAs on T1 measures, using the latent profiles as our predictor variable to test H1a-H1c. When significant differences emerged, we computed post-hoc comparisons with Holm correction. Then, we computed a structural equation model by regressing the latent profiles onto all T2 measures to test H2-H9. As pre-registered, we explored if the results changed when country of residence was included as an additional predictor in the analyses. All analyses were conducted using JASP unless otherwise noted. Anonymized data and analyses are available on our OSF page (https://osf.io/5423x).

## Results

### Baseline (T1)

#### Preliminary Analyses

At T1, most participants indicated they had oral (94.8%), vaginal (79.2%), and anal sex (78.9%). Overall descriptive statistics and correlations between measures are summarized in Table 2. As expected, participants who scored higher on prevention reported more sexual inhibition, both *p* ≤ .004, whereas participants who scored higher on promotion reported more sexual excitation, *p* < .001, and sexual communal strength, *p* < .001.

#### Sexual Regulatory Focus Profiles

Results of the latent profile analysis are summarized in Table 3. We retained the model with three latent profiles, as AIC, BIC, and SABIC values decreased (i.e., model fit improved) with the inclusion of more profiles, and the LMR test indicated that adding a fourth profile failed to significantly improve fit. Also, the entropy level of this model indicated good classification certainty (similar to the remaining models), and the percentage in the smallest profile suggested stability in the solution (similar to the model with two profiles).

**Table 3:** Latent profile analysis results

Model	Log likelihood	AIC	BIC	SABIC	Entropy	Smallest profile (%)	LMR (*p*)	Comparison	BLRT (*p*)	Comparison
1 profile	− 14,298.91	28,633.82	28,718.39	28,661.23						
2 profiles	− 13,662.41	27,380.81	27,512.37	27,423.45	.843	33	< .001	2 > 1	< .001	2 > 1
3 profiles	− 13,508.97	27,093.93	27,272.47	27,151.79	.799	29	.001	3 > 2	< .001	3 > 2
4 profiles	− 13,396.74	26,889.49	27,115.00	26,962.57	.803	20	.142	4 > 3	< .001	4 > 3

*N* = 811. AIC = Akaike’s information criterion; BIC = Bayesian information criterion; SABIC = sample size-adjusted BIC; LMR = Lo, Mendell, and Rubin test; BLRT = Bootstrapped likelihood ratio test. The LMR test and the BLRT compare the model to a model with *k−*1 profiles

**Fig. 1:**
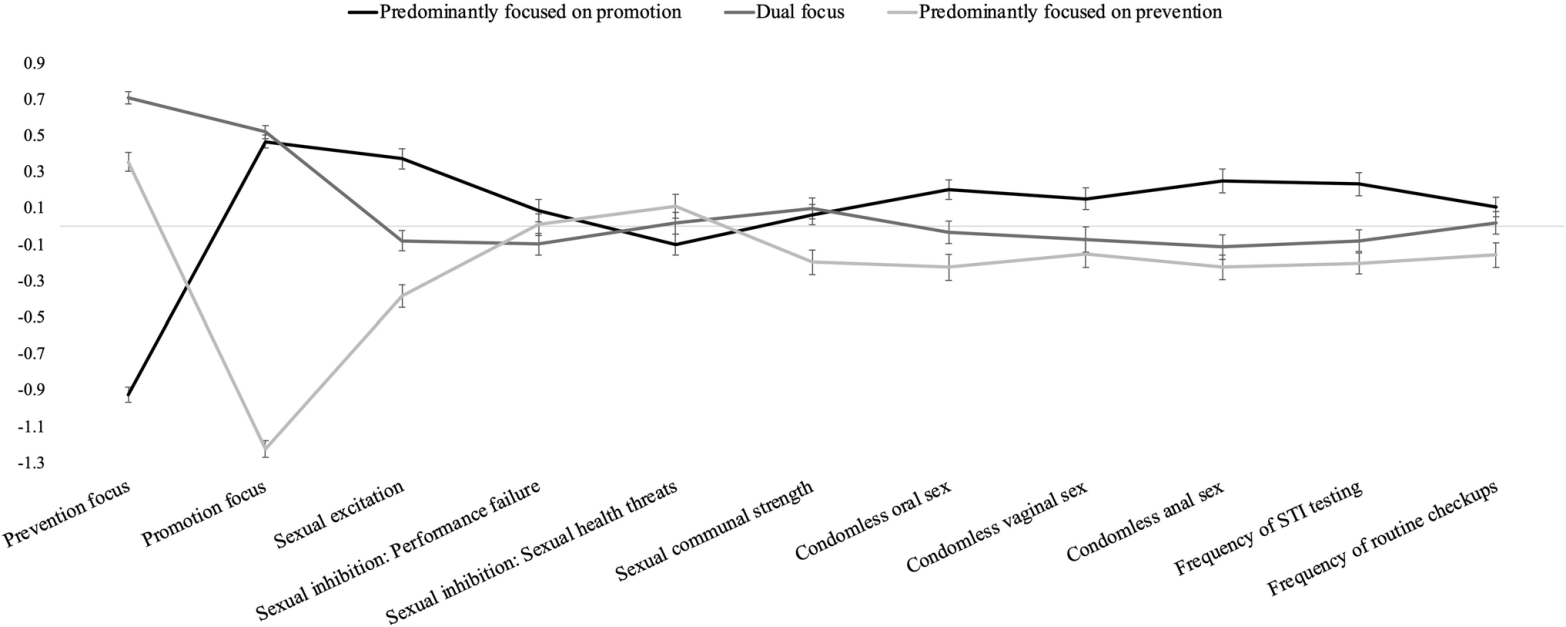
Differences between profiles in outcomes at baseline (T1). Note: Variables were standardized for visualization purposes. Error bars indicate standard errors

Marginal means and post-hoc results for each profile are summarized in Table 4, and standardized scores are depicted in Fig. 1. Participants predominantly focused on prevention (Profile 3, *n* = 232) scored higher on prevention and lower on promotion. In contrast, participants predominantly focused on promotion (Profile 1, *n* = 301) scored higher on promotion and lower on prevention. As expected, participants predominantly focused on prevention (vs. promotion) reported more sexual inhibition due to sexual health threat perceptions, *p* = .043 (H1a), whereas participants predominantly focused on promotion (vs. prevention) reported more sexual excitation, *p* < .001 (H1b), and more sexual communal strength, *p* = .007 (H1c). Replicating past research, these participants also reported having more frequent oral, *p* < .001, vaginal, *p* = .006, and anal condomless sex, *p* < .001, got tested for STI more frequently, *p* < .001, and attended more routine sexual health checkups, *p* = .007.

**Table 4:** Differences between sexual regulatory focus profiles

	Profile 1: Predominantly focused on promotion *M* (*SE*)	Profile 2: Dual focus *M* (*SE*)	Profile 3: Predominantly focused on prevention *M* (*SE*)	Post-hoc tests
*T1 (at baseline)*				
Prevention focus	3.46 (.06)	5.93 (.06)	5.40 (.07)	Profile 1 versus 2: *p* < .001 Profile 1 versus 3: *p* < .001 Profile 2 versus 3:* p* < .001
Promotion focus	5.57 (.04)	5.63 (.05)	3.56 (.05)	Profile 1 versus 2: *p* = .574 Profile 1 versus 3: *p* < .001 Profile 2 versus 3:* p* < .001
Sexual excitation	3.41 (.04)	3.06 (.05)	2.82 (.05)	Profile 1 versus 2: *p* < .001 Profile 1 versus 3: *p* < .001 Profile 2 versus 3:* p* < .001
Sexual inhibition: Performance failure	2.63 (.05)	2,48 (.05)	2.57 (.05)	Profile 1 versus 2: *p* = .070 Profile 1 versus 3: *p* = .658 Profile 2 versus 3:* p* = .446
Sexual inhibition: Sexual health threats	3.42 (.05)	3.53 (.06)	3.61 (.06)	Profile 1 versus 2: *p* = .346 Profile 1 versus 3: *p* = .043 Profile 2 versus 3:* p* = .536
Sexual communal strength	3.69 (.04)	3.71 (.04)	3.52 (.04)	Profile 1 versus 2: *p* = .908 Profile 1 versus 3: *p* = .007 Profile 2 versus 3:* p* = .002
Condomless oral sex	5.50 (.12)	5.00 (.13)	4.59 (.14)	Profile 1 versus 2: *p* = .014 Profile 1 versus 3: *p* < .001 Profile 2 versus 3:* p* = .086
Condomless vaginal sex	3.93 (.13)	3.43 (.15)	3.25 (.17)	Profile 1 versus 2: *p* = .037 Profile 1 versus 3: *p* = .006 Profile 2 versus 3:* p* = .722
Condomless anal sex	3.34 (.13)	2.54 (.15)	2.30 (.16)	Profile 1 versus 2: *p* < .001 Profile 1 versus 3: *p* < .001 Profile 2 versus 3:* p* = .495
Frequency of STI testing	3.03 (.10)	2.46 (.11)	2.23 (.12)	Profile 1 versus 2: *p* < .001 Profile 1 versus 3: *p* < .001 Profile 2 versus 3:* p* = .326
Frequency of routine checkups	2.39 (.05)	2.31 (.06)	2.14 (.06)	Profile 1 versus 2: *p* = .550 Profile 1 versus 3: *p* = .007 Profile 2 versus 3:* p* = .112
*T2 (3-month follow-up)*				
Prevention focus	3.81 (.10)	5.70 (.08)	5.40 (.11)	Profile 1 versus 2: *p* < .001 Profile 1 versus 3: *p* < .001 Profile 2 versus 3:* p* = .030
Promotion focus	5.48 (.06)	5.28 (.07)	3.90 (.08)	Profile 1 versus 2: *p* = .044 Profile 1 versus 3: *p* < .001 Profile 2 versus 3:* p* < .001
Riskier oral sex	0.03 (.07)	−0.16 (.08)	−0.17 (.12)	Profile 1 versus 2: *p* = .045 Profile 1 versus 3: *p* = .033 Profile 2 versus 3:* p* = .562
Condomless oral sex: Positive affect	3.99 (.07)	4.03 (.07)	3.56 (.12)	Profile 1 versus 2: *p* = .746 Profile 1 versus 3: *p* < .001 Profile 2 versus 3:* p* < .001
Condomless oral sex: Negative affect	1.50 (.06)	1.30 (.04)	1.58 (.08)	Profile 1 versus 2: *p* = .031 Profile 1 versus 3: *p* = .521 Profile 2 versus 3:* p* = .015
Riskier vaginal sex	0.05 (.06)	−0.25 (.07)	−0.45 (.09)	Profile 1 versus 2: *p* = .003 Profile 1 versus 3: *p* < .001 Profile 2 versus 3:* p* = .057
Condomless vaginal sex: Positive affect	4.10 (.06)	4.10 (.08)	3.88 (.10)	Profile 1 versus 2: *p* = .997 Profile 1 versus 3: *p* = .002 Profile 2 versus 3:* p* = .003
Condomless vaginal sex: Negative affect	1.49 (.06)	1.31 (.05)	1.58 (.09)	Profile 1 versus 2: *p* = .132 Profile 1 versus 3: *p* = .081 Profile 2 versus 3:* p* = .003
Riskier anal sex	−0.19 (.10)	−0.00 (.15)	−.41 (.18)	Profile 1 versus 2: *p* = .702 Profile 1 versus 3: *p* = .079 Profile 2 versus 3:* p* = .191
Condomless anal sex: Positive affect	3.94 (.13)	4.09 (.16)	3.23 (.30)	Profile 1 versus 2: *p* = .314 Profile 1 versus 3: *p* = .001 Profile 2 versus 3:* p* < .001
Condomless anal sex: Negative affect	1.52 (.09)	1.56 (.16)	1.87 (.26)	Profile 1 versus 2: *p* = .590 Profile 1 versus 3: *p* = .083 Profile 2 versus 3:* p* = .185
Reasons to have sex: Autonomous	5.10 (.08)	4.95 (.08)	4.27 (.10)	Profile 1 versus 2: *p* = .181 Profile 1 versus 3: *p* < .001 Profile 2 versus 3:* p* < .001
Reasons to have sex: Controlled	2.37 (.10)	1.91 (.08)	2.29 (.12)	Profile 1 versus 2: *p* < .001 Profile 1 versus 3: *p* = .471 Profile 2 versus 3:* p* = .013
Sexual satisfaction: Personal	5.27 (.08)	5.27 (.08)	4.57 (.10)	Profile 1 versus 2: *p* = .964 Profile 1 versus 3: *p* < .001 Profile 2 versus 3:* p* < .001
Sexual satisfaction: Activity	5.01 (.08)	4.93 (.08)	4.38 (.11)	Profile 1 versus 2: *p* = .431 Profile 1 versus 3: *p* < .001 Profile 2 versus 3:* p* < .001
Sexual health communication	2.80 (.10)	2.80 (.12)	2.76 (.13)	Profile 1 versus 2: *p* = .924 Profile 1 versus 3: *p* = .797 Profile 2 versus 3:* p* = .748
Perceived sexual health	5.30 (.09)	5.52 (.10)	4.81 (.14)	Profile 1 versus 2: *p* = .104 Profile 1 versus 3: *p* = .003 Profile 2 versus 3:* p* < .001
STI testing	16.43	14.97	9.02	Profile 1 versus 2: *p* = . 692 Profile 1 versus 3: *p* = .036 Profile 2 versus 3:* p* = .096

Values for riskier sexual activities are standardized means. For STI testing, we reported the percentage of “*Yes*” responses

A distinct group of participants showed a dual focus on prevention and promotion (Profile 2, *n* = 278). These participants reported moderate levels of sexual excitation, both *p* < .001, and more sexual communal strength than those predominantly focused on prevention, *p* = .002. Although participants with a dual focus reported engaging in condomless sex and getting STI tests less frequently than those predominantly focused on promotion, all *p* ≤ .037, both groups reported a similar frequency of routine sexual health checkup attendance, *p* = .550.

### 3-Month Follow-Up (T2)

#### Preliminary Analyses

Overall descriptive statistics and correlations between measures are summarized in Table 5.

**Table 5:** Descriptive statistics and correlations for T2 measures

	*M* (*SD*) or %	1	2	3	4	5	6	7	8	9	10	11	12	13	14	15	16	17
1. Prevention focus	4.88 (1.55)	–																
2. Promotion focus	5.01 (1.15)	− .24^***^	–															
3. Riskier oral sex	− 0.07 (0.89)	− .13^*^	.05	–														
4. Condomless oral sex: Positive affect	3.92 (0.82)	− .01	.45^***^	.10	–													
5. Condomless oral sex: Negative affect	1.45 (0.60)	− .17^**^	− .11^*^	− .08	− .21^***^	–												
6. Riskier vaginal sex	− 0.16 (0.86)	− .18^***^	.17^***^	.37^***^	.15^**^	− .07	–											
7. Condomless vaginal sex: Positive affect	4.06 (0.78)	− .04	.35^***^	.08	.64^***^	− .31^***^	.04	–										
8. Condomless vaginal sex: Negative affect	1.45 (0.62)	− .11	− .13^*^	− .14^*^	− .23^***^	.76^***^	− .21^***^	− .24^***^	–									
9. Riskier anal sex	− 0.18 (0.84)	− .07	.05	.29^**^	.16	− .14	.56^***^	.17	− .02	–								
10. Condomless anal sex: Positive affect	3.89 (0.93)	− .09	.47^***^	− .05	.68^***^	− .40^***^	− .11	.60^***^	− .27^*^	.10	–							
11. Condomless anal sex: Negative affect	1.57 (0.71)	.06	− .19	.09	− .33^**^	.49^***^	.04	− .35^**^	.58^***^	− .09	− .54^***^	–						
12. Reasons to have sex: Autonomous	4.87 (1.08)	− .11^*^	.48^***^	.12^*^	.32^***^	− .03	.15^**^	.23^***^	− .10	.15	.24^*^	.04	–					
13. Reasons to have sex: Controlled	2.19 (1.19)	− .28^***^	− .07	.03	− .22^***^	.30^***^	.05	− .21^***^	.30^***^	.00	− .27^*^	.49^***^	.03	–				
14. Sexual satisfaction: Personal	5.12 (1.05)	− .04	.50^***^	− .02	.44^***^	− .33^***^	.10^*^	.40^***^	− .35^***^	− .06	.43^***^	− .24^*^	.42^***^	− .23^***^	–			
15. Sexual satisfaction: Activity	4.85 (1.08)	− .08	.43^***^	− .00	.31^***^	− .28^***^	.11^*^	.33^***^	− .30^***^	− .03	.43^***^	− .27^*^	.36^***^	− .20^***^	.75^***^	–		
16. Sexual health communication	2.79 (1.38)	.03	.03	− .06	.01	.29^***^	− .14^**^	− .06	.27^***^	− .12	− .05	.30^**^	− .01	.14^**^	− .00	.03	–	
17. Perceived sexual health	5.25 (1.44)	− .00	.37^***^	− .05	.32^***^	− .20^***^	.02	.27^***^	− .19^***^	.15	.35^***^	− .17	.20^***^	− .20^***^	.44^***^	.42^***^	.00	–
18. STI testing	14.0	− .08	.03	.13^*^	.04	.06	.10^*^	− .01	− .02	.08	− .07	.14	.01	.08	− .01	.02	.12^*^	.05

For STI testing, we reported the percentage of “*Yes*” responses and computed point-biserial correlations. Degrees of freedom vary between 73 and 531

***p ≤ .001, **p ≤ .010, *p ≤ .050

#### Differences in Sexual Health and Well-Being Outcomes

**Fig. 2:**
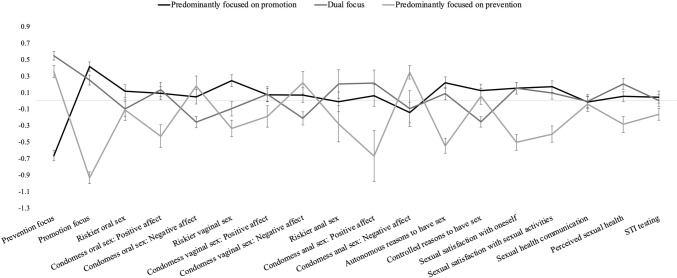
Temporal associations between sexual regulatory focus profiles (T1) and sexual health and well-being outcomes (T2). *Note:* Variables were standardized for visualization purposes. Error bars indicate standard errors

Marginal means and post-hoc results for each profile are summarized in Table 4, and standardized scores are depicted in Fig. 2. Aligned with our hypotheses, participants predominantly focused on prevention (vs. promotion) scored higher on prevention and lower on promotion (H2), both *p* < .001, reported lower levels of riskier sexual activities, both *p* ≤ .033 (except for riskier anal sex, *p* = .079; H3), experienced less positive affect when thinking about their last condomless sex experiences, all *p* ≤ .001 (but not more negative affect, all *p* ≥ .081). Unexpectedly, these participants did not endorse more sexual health communication with casual partners, *p* = .797 (H5), and perceived to have worse sexual health, *p* = .003 (H6). In contrast, participants predominantly focused on promotion (vs. prevention) endorsed more autonomous reasons for having had sex, *p* < .001 (but not more controlled reasons, *p* = .471) (H7), reported more sexual satisfaction with casual partners, both *p* < .001 (H8), and were more likely to have been tested for STIs, *p* = .036 (H9).

Participants with a dual focus did not differ from participants predominantly focused on prevention in their riskier sexual activities, all *p* ≥ .057, but reported more positive and less negative affective reactions when thinking about condomless sex, all *p* ≤ .015 (except for negative affect toward condomless anal sex, *p* = .185). Compared to participants predominantly focused on promotion, those with a dual focus endorsed autonomous reasons to a similar extent, *p* = .181, but less controlled reasons for having had sex, *p* < .001. Both groups also reported similar sexual satisfaction, *p* ≥ .431, perceived sexual health, *p* = .104, and were equally likely to have been tested for STIs, *p* = .692.

### Country Differences

At T1, participants in Spain reported more sexual excitation, *p* < .001, whereas participants in Portugal reported more frequent condomless sexual activities, all *p* ≤ .027, got tested for STIs more frequently, *p* < .001, and attended more routine sexual health checkups, *p* = .015. At T2, participants in Spain reported more negative affect when recalling their latest oral condomless sex encounters, *p* = .009, whereas participants in Portugal reported more sexual satisfaction, both *p* ≤ .013. Despite these a priori differences, the overall pattern of results reported above remained unchanged.

## Discussion

Results of a longitudinal study with samples from two European countries showed how differences in sexual regulatory focus determined responses, behaviors, and experiences in casual sex encounters. We used a person-centered approach to determine profiles based on the pattern of responses, while accounting for individual variability (for a similar approach, see Rodrigues et al., 2024a, 2024b, 2024c), instead of relying on artificial classifications (e.g., difference scores). Taken together, our results are aligned and extend the regulatory focus framework (e.g., Evans-Paulson et al., 2022; Rodrigues et al., 2022), extend other theoretical frameworks (Janssen & Bancroft, 2023; Muise et al., 2013), and highlight differences between sexual activities (e.g., Ballester-Arnal et al., 2022).

Based on our findings, people predominantly focused on prevention (i.e., driven by safety in sex) tend to be more restricted in their sexual activity to avoid potential health problems, but are also likely to have worse casual sex experiences later on. As expected, participants predominantly focused on prevention (vs. promotion) reported higher sexual inhibition related to risk awareness (and lower sexual excitation), reported having enacted fewer riskier sexual activities three months later, felt worse when condomless sex occurred, and reported being less sexually satisfied. These findings are aligned with past research, as one of the potential consequences of a safety-driven decision-making process is having less satisfying sexual experiences with casual partners, and a less positive approach toward sex and sexuality (Evans-Paulson et al., 2022; Rodrigues et al., 2024a, 2024b, 2024c). Contrary to our expectations, participants predominantly focused on prevention (vs. promotion) were not more likely to talk about sexual health with casual partners despite their efforts to avoid sexual risks, and actually reported worse sexual health later on. These findings might be explained by situational and interpersonal factors within the casual sex encounter. For example, individuals predominantly focused on prevention may not feel comfortable addressing sexual health practices in certain situations (e.g., unexpected sex with a stranger) or specific activities (e.g., oral sex), or may decide to have condomless sex under certain circumstances (e.g., when the partner has negative STI testing results; Rodrigues et al., 2024a, 2024b, 2024c). Moreover, although these individuals tend to report having more control over condom use (Rodrigues et al., 2022), this may not necessarily translate into actual control. For example, external pressures, such as condom negotiation strategies employed by casual partners (e.g., Davis et al., 2014; Noar et al., 2002), may compromise their ability to use condoms consistently, which could lead them to experience more distress or perceive worse sexual health outcomes when condomless sexual encounters do occur.

Our findings also suggest that people predominantly focused on promotion (i.e., driven by pleasure) tend to take more risks in casual sexual encounters and are likely to attain (and offer) more sexual rewards with casual partners. As expected, participants predominantly focused on promotion (vs. prevention) reported higher sexual arousal (and less sexual inhibition), more interest in attending to their partners’ sexual needs, and more inconsistent condom use across different sexual activities, but they also got tested for STIs and attended routine sexual health checkups more often. Three months later, these participants were more likely to have enacted riskier sexual behaviors (i.e., more condomless sex with a higher number of partners) and felt better about their condomless sexual activities, engaged in casual sex for hedonistic reasons, were more satisfied with their casual sex lives, and were more likely to have been tested for STIs. Past studies have already suggested that individuals under a predominant promotion focus may address potential health problems by enacting surveillance practices (Rodrigues et al., 2023), which may explain why these participants got tested for STIs and reported better sexual health three months later. Hence, having a predominant focus on promotion does not necessarily equate to being irresponsible in casual encounters, but rather weighing the immediate sexual rewards relative to the health costs before deciding the course of action. This argument is supported by the evidence that condom use intentions decrease the longer individuals predominantly focused on promotion have to wait for a condom to be available before having casual sex in a hypothetical scenario, particularly when the risk of STI infection is low (Rodrigues, 2023).

Extending past studies, we identified for the first time a third sexual regulatory focus profile. People with a dual focus tend to be cautious with their sexual behaviors and act in ways to protect their sexual health (e.g., more protected sexual activities, similar to participants predominantly focused on prevention), but at the same time want to attain and offer sexual rewards with casual partners (e.g., more sexual satisfaction, similar to participants predominantly focused on promotion). These findings highlight the complexities and nuances of sexual regulatory focus, indicating that some individuals can simultaneously balance risk prevention and pleasure promotion in casual sex encounters. Unlike individuals with a single predominant focus, those with a dual focus seemed to successfully integrate both motivations to their advantage and navigate casual sex encounters with a combination of risk caution and pleasure-seeking.

### Limitations and Future Studies

We must acknowledge some limitations and offer suggestions for future studies. Some of our measures had low reliability indices, which can introduce measurement error and potentially impact our findings. Although similar reliability indices have been reported in past research (e.g., Hogue et al., 2019; Shoikhedbrod et al., 2023; Sierra et al., 2024), future studies could consider revising the measures and conducting validation studies across cultural contexts. Also, our samples were WEIRD (Western, educated, industrialized, rich, and democratic), which can potentially impact the generalizability of our results. Although sexual regulatory focus has been extended to other Western cultural contexts (e.g., Germany, United States; Evans-Paulson et al., 2022; Rodrigues, 2022, 2023), future studies could consider recruiting a more diverse sample of individuals. Lastly, our study may have been underpowered given the T2 sample size. Although we took a conservative approach to power analyses and have confidence in our findings, future studies could consider replicating our findings with a larger sample of participants.

Extending our current findings, future studies could consider examining whether sexual regulatory focus interplays with other interpersonal and intergroup variables. Based on our current findings, particular attention should be given to individuals with a dual sexual regulatory focus profile, as they can offer relevant insights. For example, researchers could examine if individuals enact different condom negotiation strategies (i.e., condom avoidance vs. condom approach) with casual partners. Researchers could also consider examining if sexual experiences and health practices vary according to power dynamics, social expectations, or normative pressures in the sexual encounter. Lastly, our results suggest that sexual regulatory focus profiles were consistent over time. However, the question remains as to whether participants can change their predominant sexual regulatory focus—either momentarily or permanently—depending on certain contextual cues. For example, future studies could explore if and under which conditions individuals with a predominant promotion profile change their riskier sexual behaviors (e.g., using condoms to protect others after receiving a positive STI diagnosis), or if and under which conditions individuals with a predominant prevention profile have more positive sexual experiences (e.g. after partners test negative for STIs and the risk of infection is objectively lower).

### Conclusions

Using a novel approach within the regulatory focus framework, this longitudinal study showed how distinct sexual regulatory focus profiles influenced sexual activity patterns and experiences, sexual satisfaction, and sexual safety practices with casual sex partners over three months. These findings underscore the importance of recognizing the specificity and diversity within sexual regulatory focus to better inform efforts aimed at fostering sexual health and well-being for all.

## Supplementary Information

Below is the link to the electronic supplementary material.

Supplementary file1 (DOCX 38 KB)

## Data Availability

Materials and anonymized data that support these findings (https://osf.io/5423x) and all studies, materials, and outputs related to the Prevent2Protect project (https://osf.io/rhg7f) are publicly available.
