# Supplementary material for: Longitudinal Associations Between Sexual Regulatory Focus and Sexual Health and Well-Being
Source: Arch Sex Behav. 2026 Apr 18;55(3):1193–206. doi: 10.1007/s10508-026-03427-z (PMC13194272; doi:10.1007/s10508-026-03427-z)
Supplement: Supplementary file 1 — Supplementary file1 (DOCX 38 KB) [file 10508_2026_3427_MOESM1_ESM.docx]

**Supplementary Materials**

**S1**

*Fit Indexes from the Confirmatory Factor Analyses*

| Measure | df | *χ*^2^ (*p*) | SRMR | TLI | CFI | RMSEA | λ (*p* values) |
| --- | --- | --- | --- | --- | --- | --- | --- |
| Regulatory focus in sexuality (T1) | 26 | 91.52 (< .001) | .03 | .96 | .97 | .06 | ≥ .41 (all < .001) |
| Sexual inhibition/sexual excitation (T1) | 70 | 254.05 (< .001) | .06 | .90 | .92 | .06 | ≥ .25 (all < .001) |
| Regulatory focus in sexuality (T2) | 26 | 118.15 (< .001) | .04 | .92 | .94 | .08 | ≥ .49 (all < .001) |
| Positive/negative affect – Oral sex (T2) | 25 | 49.37 (.003) | .04 | .96 | .97 | .06 | ≥ .38 (all < .001) |
| Positive/negative affect – Vaginal sex (T2) | 25 | 57.14 (< .001) | .05 | .95 | .97 | .07 | ≥ .30 (all < .001) |
| Positive/negative affect – Anal sex (T2) | 25 | 42.25 (.017) | .06 | .93 | .95 | .09 | ≥ .32 (all ≤ .003) |
| Sexual satisfaction (T2) | 51 | 255.56 (< .001) | .04 | .91 | .93 | .09 | ≥ .51 (all < .001) |

*Note*. df = Degrees of freedom; SRMR = Standardized root mean square residual; TLI = Tucker-Lewis fit index; CFI = Comparative fit index; RMSEA = Root mean square error of approximation; λ = Standardized regression coefficients. CFA results for the PANAS scales showed that λ values for item 5 from the positive affect subscales (i.e., “Alert”) were non-significant (all *p* ≥ .405) and therefore this item was excluded from the analyses.

**S2**

*Results of the Exploratory Factor Analyses*

| Measure | KMO | Number of factors | Explained variance | Rotated factor loadings |
| --- | --- | --- | --- | --- |
| Sexual communal strength (T1) | .73 | 1 | 31% | - |
| Reasons to have sex (T2) | .64 | 2 | 37% | - |
| Factor 1: Controlled | - | - | 21% | ≥ .44 |
| Factor 2: Autonomous | - | - | 16% | ≥ .46 |
| Sexual health communication (T2) | .83 | 1 | 42% | - |

*Note*. Items 4 and 8 form the Sexual Motivation Scale were excluded from the analysis due to factor loadings. To have a more inclusive scale, two items from the Health Protective Sexual Communication Scale were removed: “Talked about whether you or a new sex partner ever had homosexual experiences” and “Talked to a new sex partner about birth control before having sex for the first time”.
